# Exploring *Klebsiella pneumoniae* in Healthy Poultry Reveals High Genetic Diversity, Good Biofilm-Forming Abilities and Higher Prevalence in Turkeys Than Broilers

**DOI:** 10.3389/fmicb.2021.725414

**Published:** 2021-09-07

**Authors:** Fiona V. Franklin-Alming, Håkon Kaspersen, Marit A. K. Hetland, Ragna-Johanne Bakksjø, Live L. Nesse, Thongpan Leangapichart, Iren H. Löhr, Amar A. Telke, Marianne Sunde

**Affiliations:** ^1^Section for Microbiology, Department of Analysis and Diagnostics, Norwegian Veterinary Institute, Oslo, Norway; ^2^Research Section Food Safety and Animal Health, Department of Animal Health and Food Safety, Norwegian Veterinary Institute, Oslo, Norway; ^3^Department of Medical Microbiology, Stavanger University Hospital, Stavanger, Norway; ^4^Department of Biological Sciences, Faculty of Mathematics and Natural Sciences, University of Bergen, Bergen, Norway

**Keywords:** *Klebsiella pneumoniae*, poultry, biofilm, antimicrobial resistance, hypervirulence

## Abstract

*Klebsiella pneumoniae* is a well-studied human pathogen for which antimicrobial resistant and hypervirulent clones have emerged globally. *K. pneumoniae* is also present in a variety of environmental niches, but currently there is a lack of knowledge on the occurrence and characteristics of *K. pneumoniae* from non-human sources. Certain environmental niches, e.g., animals, may be associated with high *K. pneumoniae* abundance, and these can constitute a reservoir for further transmission of strains and genetic elements. The aim of this study was to explore and characterize *K. pneumoniae* from healthy broilers and turkeys. A total of 511 cecal samples (broiler *n* = 356, turkey *n* = 155), included in the Norwegian monitoring program for antimicrobial resistance (AMR) in the veterinary sector (NORM-VET) in 2018, were screened for *K. pneumoniae* by culturing on SCAI agar. *K. pneumoniae* was detected in 207 (40.5%) samples. Among the broiler samples, 25.8% were positive for *K. pneumoniae*, in contrast to turkey with 74.2% positive samples (*p* < 0.01). Antibiotic susceptibility testing was performed, in addition to investigating biofilm production. Whole genome sequencing was performed on 203 *K. pneumoniae* isolates, and analysis was performed utilizing comparative genomics tools. The genomes grouped into 66 sequence types (STs), with ST35, ST4710 and ST37 being the most prevalent at 13.8%, 7.4%, and 5.4%, respectively. The overall AMR occurrence was low, with only 11.3% of the isolates showing both pheno- and genotypic resistance. Genes encoding aerobactin, salmochelin or yersiniabactin were detected in 47 (23.2%) genomes. Fifteen hypervirulent genomes belonging to ST4710 and isolated from turkey were identified. These all encoded the siderophore virulence loci *iuc5* and *iro5* on an IncF plasmid. Isolates from both poultry species displayed good biofilm-forming abilities with an average of OD_595_ 0.69 and 0.64. To conclude, the occurrence of *K. pneumoniae* in turkey was significantly higher than in broiler, indicating that turkey might be an important zoonotic reservoir for *K. pneumoniae* compared to broilers. Furthermore, our results show a highly diverse *K. pneumoniae* population in poultry, low levels of antimicrobial resistance, good biofilm-forming abilities and a novel hypervirulent ST4710 clone circulating in the turkey population.

## Introduction

*Klebsiella pneumoniae* is an opportunistic pathogen that is on the short list of common causes of nosocomial infections, together with five other bacteria, collectively referred to as the ESKAPE-pathogens (Santajit and Indrawattana, 2016). *Klebsiella* spp. from humans have undergone extensive research to characterize the bacteria’s pheno-and genotypes. There are many different species and subspecies of *Klebsiella*, but *K. pneumoniae* is clinically the most important species within this genus (Wyres and Holt, 2018). *K. pneumoniae* can be further divided into seven subspecies, together comprising the *K. pneumoniae* species complex (KpSC) (Rodrigues et al., 2019). Some *K. pneumoniae* lineages are associated with high levels of antimicrobial resistance (AMR), e.g., sequence type (ST) 258, while other lineages, e.g., ST23, are considered hypervirulent (Struve et al., 2015). *K. pneumoniae* may cause infections such as urinary tract infections, pneumonia, septicemia and liver abscesses (Ko et al., 2002). Resistance toward clinically relevant antimicrobials is generally not observed in hypervirulent strains, and antimicrobial resistant strains are rarely hypervirulent (Lee et al., 2017). However, convergent strains of *K. pneumoniae* have been observed, and are increasingly being reported worldwide (Wyres et al., 2020). Other factors than AMR, such as biofilm production, can contribute to increased survival and emergence of *K. pneumoniae*. Biofilm-lifestyle provides protection against disinfectants and antibiotics, and facilitates persistence in production environments (Vestby et al., 2009; Osland et al., 2020). In addition, both horizontal gene transfer and mutations occur at higher rates within biofilms than in planktonic cultures (Nesse and Simm, 2018). This may be a cause for concern, as this could contribute to the evolution of more resistant and resilient bacteria in the livestock production and food industry. The importance of biofilm formation by *Klebsiella* isolates originating from humans has previously been identified in relation to biofilms on e.g., surgical implants and catheters (Francolini et al., 2017). However, there is a lack of knowledge to whether *Klebsiella* from animals or the environment have the same biofilm forming abilities.

Many studies involving *K. pneumoniae* focus on clinical isolates from humans. Little is known about the occurrence of *K. pneumoniae* in healthy animals, and whether lineages or genetic elements associated with human infections are present in the animal population. Our understanding of the spread of pathogens from animals to humans is of outmost importance as animals may act as reservoirs for human clinical *K. pneumoniae* infection. Therefore, the aim of this study was to characterize *K. pneumoniae* isolates from broiler and turkey populations in Norway by AMR screening and whole genome sequencing. Additionally, the population structure of the isolates was determined to identify any shared lineages and genetic elements associated with human *K. pneumoniae*.

## Materials and Methods

### Isolation and Identification of *Klebsiella pneumoniae*

From January to December 2018, cecal samples from broilers and turkeys, included in the Norwegian monitoring program for AMR in the veterinary sector (NORM-VET) were screened for *K. pneumoniae*. Further information regarding the sampling is included in the annual NORM/NORM-VET report (NORM/NORM-VET, 2018). Ten cecal samples were collected from each poultry flock at slaughter, and these samples were pooled into one from either broiler (*n* = 356) or turkey (*n* = 155), resulting in a total of 511 pooled samples. To screen for *K. pneumoniae*, fresh sample material was spread directly onto Simmons citrate agar with 1% inositol (SCAI, Oxoid) and incubated aerobically at 37 ± 1.0°C for 48 h. Presumptive *K. pneumoniae* isolates were subcultured and confirmed by using matrix assisted laser desorption time of flight mass spectrometry (MALDI-TOF MS, version 4.1.70 PYTH). One confirmed *K. pneumoniae* isolate per sample was stored at −80°C until further analyses.

### Antimicrobial Susceptibility Testing

The disk diffusion method described by the European committee for antimicrobial susceptibility testing (EUCAST) was used to determine the antimicrobial susceptibility of the isolates (Matuschek et al., 2014). The following antimicrobial agents were included in the panel: cefotaxime 5 μg, ceftazidime 10 μg, cefepime 30 μg, ampicillin 10 μg, ciprofloxacin 5 μg, nalidixic acid 30 μg, tetracycline 30 μg, tigecycline 15 μg, trimethoprim 5 μg, trimethoprim-sulfamethoxazole 25 μg, streptomycin 10 μg, gentamicin 10 μg, meropenem 10 μg and chloramphenicol 30 μg. Clinical breakpoints recommended by EUCAST^1^ were used to classify isolates as resistant or susceptible. An in-house breakpoint from the Norwegian Veterinary institute was used to determine resistance for streptomycin, and for tigecycline and nalidixic acid the EUCAST recommended epidemiological cut-off value for *K. pneumoniae* and *Escherichia coli*, respectively, were used. Isolates classified as resistant toward antimicrobial agents other than ampicillin were subjected to minimum inhibitory concentrations (MICs) determination using the EUVSEC plate from SensiTitre (TREK Diagnostics, Ltd.). *Escherichia coli* ATCC 25922 and a previously characterized multidrug-resistant *K. pneumoniae* (designated 2013-01-5243) isolate were used as controls in both assays.

### Whole Genome Sequencing

A total of 203 *K. pneumoniae* isolates, confirmed by MALDI-TOF, were subjected to whole genome sequencing. DNA extraction was performed with the MagNA Pure 96 system (Roche Applied Science) with the Viral NA Small volume Kit following the Pathogen Universal 200 4.0 purification protocol. To prepare the DNA for sequencing, an Illumina DNA Flex prep kit (Illumina Inc.) was used, and the sequencing kit utilized was Illumina Miseq Reagent V3 kit (600 cycle), generating 300 bp paired-end reads using the Illumina MiSeq platform.

### Assembly, Annotation and Pan-Genome Analysis

FastQC^2^ version 0.11.9 was used for quality control of the raw reads, followed by Trim Galore^3^ version 0.6.4 for adapter removal and trimming of low-quality nucleotides. The trimmed reads were assembled with Unicycler (Wick et al., 2017) version 0.4.8, using default settings. Kleborate (Lam et al., 2020) version 2.0.4 was then used on the assemblies to obtain basic assembly statistics, verify bacterial species, determine STs, identify capsule types, and to identify resistance- and virulence genes. Hypervirulence has previously been defined according to Huynh et al., “Hypervirulent Kp were defined as isolates harboring at least one of the genes *rmpA* and *rmpA2*, and/or at least one complete gene cluster among *iucABCD-iutA* (aerobactin) and *iroBCDN* (salmochelin).” (Huynh et al., 2020). This definition was used in the current study. Prokka (Seemann, 2014) version 1.14.5 was used to annotate the quality-controlled assemblies, using the *K. pneumoniae* NTUH-K2044 (accession number NC_012731) as the priority reference for the annotation. The GFF-files from Prokka were used as input to Panaroo (Tonkin-Hill et al., 2020) version 1.2.2 to predict the pangenome. Additionally, to identify possible contamination and potential outliers in the data, the panaroo_qc script was used. Core genes were defined as those present in at least 99% of the genomes. The predicted core genes were aligned using PRANK (Löytynoja, 2014) version 170427 as an option in Panaroo.

### Phylogenetic Analysis

Seqkit (Shen et al., 2016) version 0.2.0 was used to deduplicate the core gene sequence alignment from Panaroo. IQ-Tree (Nguyen et al., 2015) version 1.6.12 was used to generate a maximum likelihood phylogeny from the deduplicated core gene alignment, with ultrafast bootstrapping (Hoang et al., 2018) and model finder plus (Kalyaanamoorthy et al., 2017). The resulting tree was visualized in R (R Core Team, 2021) version 4.0.2 using the ggtree package (Yu et al., 2017, 2018; Yu, 2020) version 2.2.0. Hypervirulent genomes were further analyzed with a deeper phylogenetic analysis. First, core genome SNPs were called by using ParSNP (Treangen et al., 2014) version 1.2. Then, Harvesttools was used for file conversion, followed by deduplication with seqkit. Gubbins (Croucher et al., 2015) version 2.4.1 was used to remove recombinant sites in the alignment, using the GTRGAMMA model. Lastly, IQ-Tree was used to generate a maximum-likelihood phylogenetic tree in a similar manner as above, using the masked alignment. The resulting trees were visualized in a similar manner as above.

### Long-Read Sequencing and Plasmid Assembly

An isolate containing an IncF plasmid of interest was subjected to long-read sequencing (Isolate ID 2018-01-1097). DNA was extracted with MagAttract HMQ DNA kit (Qiagen), and the resulting DNA extract was barcoded with a Rapid barcoding kit (SQK-RBK004). The sequencing was performed on a MinION flowcell (FLO-MIN106). High accuracy basecalling was done with Guppy^4^ version 3.4.5, and demultiplexing was done with qcat^5^ version 1.1.0 with the –trim option and guppy mode. The long reads were corrected using Canu (Schumer et al., 2017) version 1.9. Reads shorter than 1,000 bp were filtered out using Filtlong^6^ version 0.2.0. The corrected and filtered long reads were then assembled together with short reads to generate a hybrid assembly, using Unicycler with default settings. The circularized plasmid sequence was extracted and subsequently annotated using Prokka as described above. The annotations were manually inspected in Artemis (Carver et al., 2012) version 18.1.0 and visualized in comparison to two previously published virulence plasmids using EasyFig (Sullivan et al., 2011) version 2.2.5. A previously published virulence plasmid was used as one of the references in the figure (accession number CP006635), and the other reference plasmid was identified using Mash (Ondov et al., 2016) version 2.2.2.

### Biofilm Forming Abilities of *Klebsiella pneumoniae*

The isolates biofilm producing capabilities were tested in microtiter plates, as previously described by Nesse et al. (2014). Luria-Bertani (LB) broth (Merc KGaA) was inoculated with fresh bacteria to an OD_595_ of approximately 1.0. Further 30 μl of the bacterial culture and 100 μl of LB without NaCl (LB^WO^/NaCl; Bacto-tryptone 19 g/liter, yeast extract 5 g/liter) was manually added using an automatic multipipette (Thermo Fisher Scientific) to each well in a 96-well Nunc^TM^ Nunclon^TM^ –plate (Nunc A/S) with lid and flat bottom and incubated aerobically at 20.0 ± 1.0°C for 48 h.

Each isolate was tested in triplicate, and each test was performed three times. On each test, 130 μl of LB^WO^/NaCl was used as control. After the incubation period the bacterial growth was measured using a spectrophotometer at OD_595_ before continuing with cleaning, staining with 1% crystal-violet (Sigma-Aldrich) and dissolving the biofilm with an ethanol:acetone (70:30) mixture (Prolab).

The procedure described by Nesse et al. (2014) was used to calculate the biofilm-forming abilities of each isolate. Briefly, for each triplicate set, the median value was calculated. Then the median value of the controls on the respective plate was subtracted. Since the test was performed three times, the mean of the three median values for each sample was calculated and used in further analysis. A cut-off value was used to determine the biofilm-forming abilities of each isolate as described by Stepanovic et al. (2000) with a minor modification. In short, the median of each control triplicate per plate was calculated. Then, the mean and standard deviation for all these medians were calculated for the entire experiment. The cut-off value was then defined as three standard deviations above this mean value.

### Statistical Analysis

All statistical analyses were performed in R version 4.0.2. A chi-squared test was used to determine if there was a significant difference in the occurrence of *K. pneumoniae* between the samples from broilers and turkeys. A Welch two sample *t*-test was used to determine if there was a significant difference between the measured biofilm-forming capabilities of isolates from broiler vs. turkey.

## Results

### Prevalence of *Klebsiella pneumoniae*

Overall, growth of *Klebsiella* spp. was detected in 213 (41.7%) of the 511 pooled samples. Of the 213 isolates, MALDI-TOF confirmed 207 isolates as *K. pneumoniae* (97.2%), five as *K. oxytoca* (2.3%), and one as *K. variicola* (0.5%). Species other than *K. pneumoniae* were excluded from further analysis. For the 356 samples originating from broilers, 92 (25.8%) were culture positive for *K. pneumoniae*, while the 155 samples originating from turkeys had 115 (74.2%) culture positive samples. The prevalence of *K. pneumoniae* in samples from broilers was significantly lower compared to samples from turkeys [χ^2^(1, *N* = 511) = 103.38, *p* < 0.01].

Four of the 207 *K. pneumoniae* isolates were lost in connection with storage, therefore 203 isolates were subjected to further analysis.

### Resistance Profiles for *Klebsiella pneumoniae* Isolated From Poultry

Antimicrobial susceptibility testing of the 203 isolates showed that 23 isolates (11.3%) were resistant to one or more of the included antimicrobials, excluding ampicillin (Table 1). Of the 23 resistant isolates, 17 originated from turkeys and six from broilers. Overall, 15.8% of the turkey isolates and 6.6% of the broiler isolates expressed resistance. Resistance to tetracycline occurred most frequently, identified in 16 isolates (7.9%). One isolate from broiler expressed resistance to four antimicrobial agents: tetracycline, trimethoprim, ciprofloxacin and sulfamethoxazole, whereas one isolate from turkey was resistant to both sulfamethoxazole and tetracycline. The remaining isolates expressed only resistance to one class of antimicrobials, excluding ampicillin. Among the 23 phenotypically resistant isolates, corresponding resistance genes were identified in all isolates and for all antimicrobials (Table 1).

**TABLE 1:** Geno-and phenotypic resistance found in *K. pneumoniae* isolated from broiler and turkey.

ST (n)	Source	Genotype	AMR phenotype	MIC value (mg/l)
ST35 (1)	Turkey	*sul2 tetB*	SMX TET	>1,024 (SUL) >64 (TET)
ST35 (5)	Turkey	*sul2*	SMX	>1,024 (SUL)
ST39 (1)	Turkey	*catA1*	CHL	>128 (CHL)
ST463 (2)	Broiler	*tetA*	TET	>64 (TET)
ST3069 (5)	Turkey	*tetB*	TET	>64 (TET)
ST1779 (4)	Turkey	*tetD*	TET	>64 (TET)
ST1823 (1)	Turkey	*tetD*	TET	>64 (TET)
ST2441 (3)	Broiler	*aada2 sul1*	SMX	>1,024 (SUL)
ST2458 (1)	Broiler	*dfrA1 sul1 tetA qnrS1*	TMP SUL TET	>32 (TMP) >1,024 (SUL) >64 (TET)

*CHL, chloramphenicol; TET, tetracycline; TMP, trimethoprim; SMX, sulfamethoxazole.*

### Virulence Gene Screening

Known virulence genes were detected in 47 of the 203 isolates (23.2%). The siderophore locus yersiniabactin was identified in 45 (22.2%) genomes, salmochelin in 16 (7.9%) and aerobactin in 15 (7.4%) of the genomes, while *rmpA*-genes were not found. Among the 45 genomes containing yersiniabactin, seven different *ybt* lineages were identified. Major lineages included *ybt16* in 15 genomes (33.3%) and *ybt14* in 12 (27.9%), associated with ICE*Kp12* and ICE*Kp5*, respectively. As for aerobactin and salmochelin, only the *iuc5* and *iro5* lineages were identified, in addition to one salmochelin of unknown lineage. All 15 *iuc5* and *iro5* positive isolates belonged to ST4710, the isolate with salmochelin of unknown lineage grouped into ST4106.

### Capsule Typing

Among the 203 genomes, 182 were categorized into 41 different K-loci, while the K-locus of the remaining 21 genomes could not be determined. The most frequent K-locus types included KL21 (15.8%), KL22 (11.8%), and KL14 (9.4%). KL1 and KL2 were not found. All ST4710 isolates had K-type KL21.

### Species Identification and ST Determination

**FIGURE 1:**
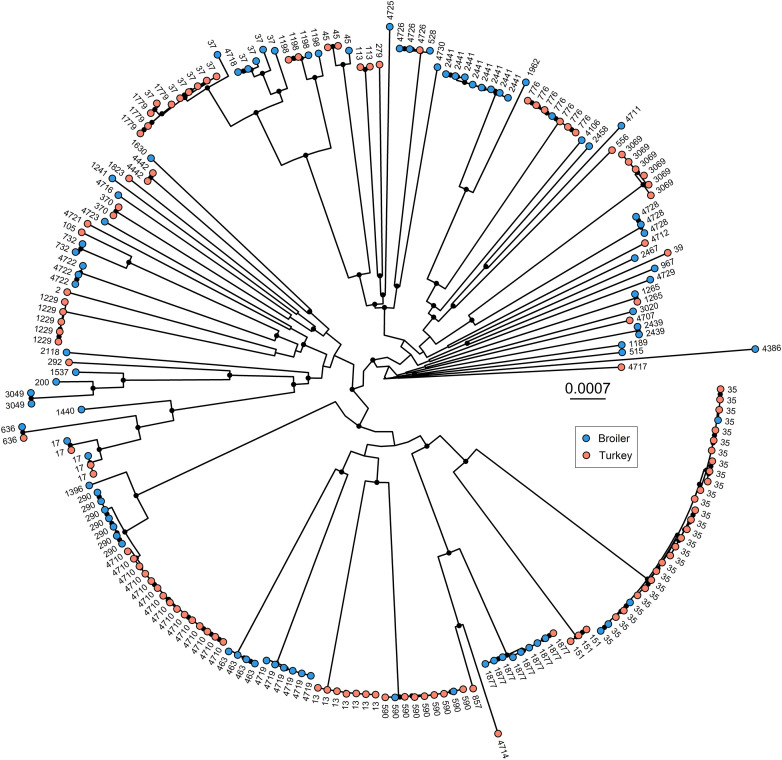
Maximum likelihood phylogenetic tree based on concatenated core genes (*n* = 4178), generated in IQ-Tree. Black dots on the internal nodes represent the bootstrap represent accepted bootstrap values (> = 95). Colors on tips represent host species, and tip labels represent sequence types. Evolutionary model: GTR + F + R10.

All the 203 isolates were confirmed as *K. pneumoniae sensu stricto* (hereafter *K. pneumoniae*) using Kleborate. A total of 66 STs were detected, where 44 and 33 different STs were identified in broilers and turkeys, respectively. Eleven STs were shared between both poultry species (Figure 1). Overall, three major STs were identified; 28 genomes grouped into ST35 (13.8%), 15 into ST4710 (7.4%), and 11 into ST37 (5.4%). ST4710 was only present in samples from turkeys (Figure 1).

### Pangenome- and Phylogenetic Analysis

The pangenome analysis identified 14,718 unique genes among all included genomes. Of these, 4,178 genes were regarded as core genes. A total of 211,085 SNPs were detected in the core genes, which were used to generate the core gene tree (Figure 1). STs clustered in concordance with the phylogenetic structure, the only exception being ST37, which was regarded as a polyphyletic ST.

### Hypervirulent ST4710 Isolates

**FIGURE 2:**
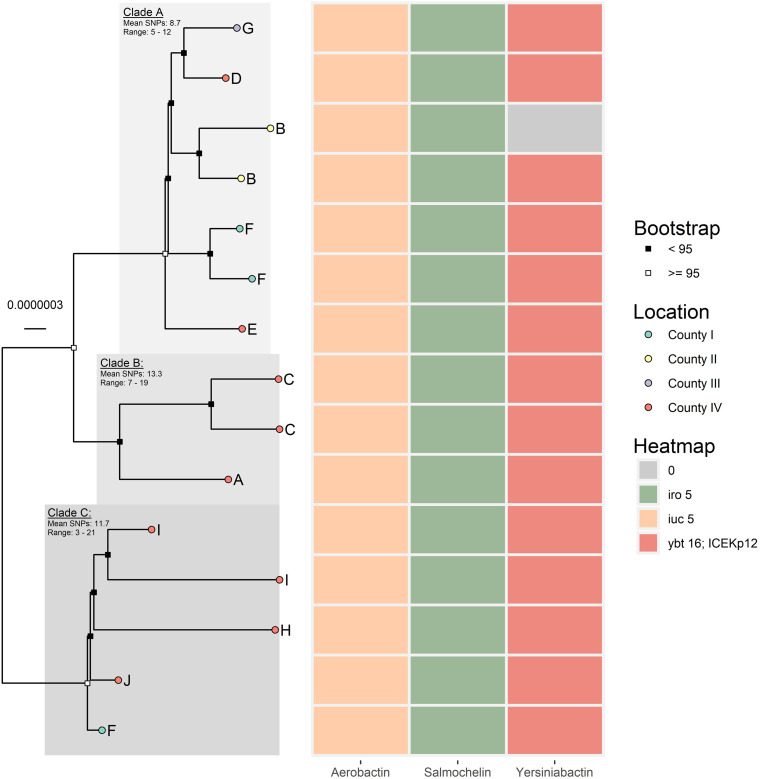
Core genome maximum likelihood tree of the 15 ST4710 *K. pneumoniae* isolates, generated in IQ-Tree. Core genome average coverage among all included isolates were 95.6%. Evolutionary model: HKY + F + I. Letters A-I represent different farms. The heat map presents the presence/absence of aerobactin, salmochelin, and yersiniabactin for each respective isolate.

A deeper phylogenetic analysis was performed on ST4710 (Figure 2). ParSNP detected in total 149 SNPs among the 15 ST4710. After masking recombinant areas, 104 SNPs were used to reconstruct the phylogeny. Overall, the ST4710 genomes were highly similar, with a core genome average coverage of 95.6% and an average SNP distance of 20.7. The isolates in this clade originated from turkey from four different counties in Norway and clustered into three distinct clades. Isolates from county IV was present in all three clades, while isolates from county II was only present in Clade A (Figure 2).

**FIGURE 3:**
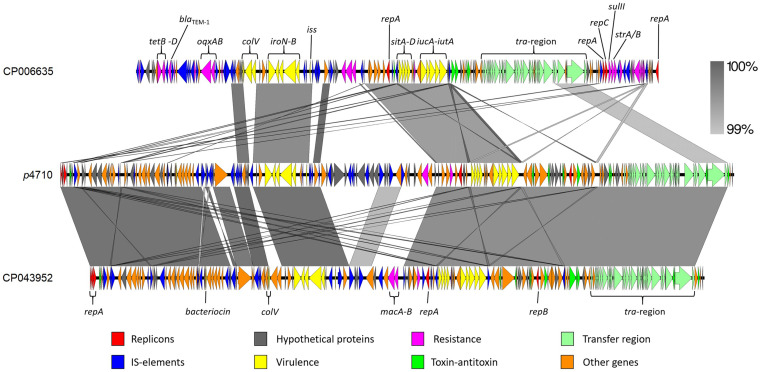
Comparison of the p4710 *K. pneumoniae* IncF plasmid from the current study (center), and the two reference plasmids CP006635 *E. coli* of porcine origin and CP043952 *E. coli* originating from broiler. Both *E. coli* were isolated in China.

An IncFII plasmid (208,340 bp) containing *iro5* and *iuc5* was detected in all 15 *K. pneumoniae* ST4710, and designated p4710. The linear map of plasmid p4710 in comparison with the most similar plasmids from GenBank is shown in Figure 3. p4710 was similar to *iro5* and *iuc5*-harboring plasmids from *E. coli* originating from pigs and broilers in China. Plasmid p4710 is a multireplicon plasmid that harbored three replicon systems (repFII, repFIB and repFIC) with subtype F18; A-:B1. A total of 221 open reading frames were predicted. Multiple toxin-antitoxin (TA)-based addiction systems (i.e., *relE/parE, phd/yefM, vapC, ccdA/ccdB*, and *hok/sok*) and plasmid partitioning systems, *parAB* and *psiAB*, which are important for plasmid maintenance, were found upstream of the *tra* region. A complete *tra* region which encodes for the transfer component was also identified in p4710.

### Determination of Biofilm Forming Abilities of *Klebsiella pneumoniae*

**FIGURE 4:**
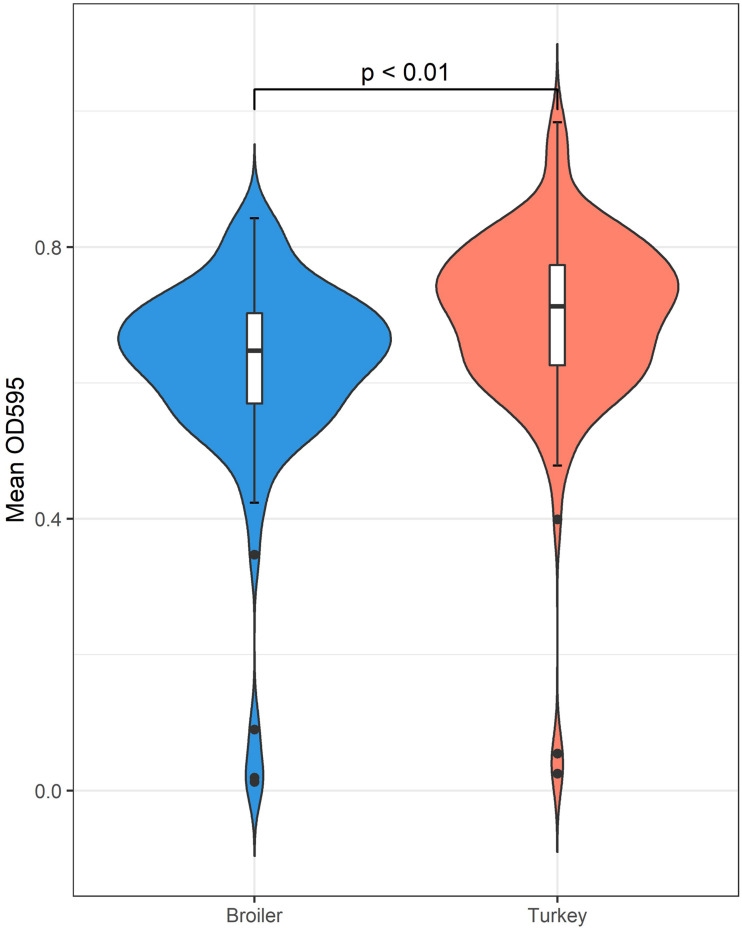
The biofilm forming abilities of *K. pneumoniae* measured by OD_595_ indicates a statistically significant difference favoring isolates originating from turkey samples.

In total, 198 of the 203 isolates (97.5%) were regarded as biofilm producers, based on the calculated cut-off value of OD_595_ 0.099. Of the five non-biofilm producers, three were from broilers and two were from turkeys. The average OD_595_ for all isolates was OD_595_ 0.66 (SD ± 0.14). The average OD_595_ value for broiler and turkey isolates were OD_595_ of 0.62 and OD_595_ 0.69, respectively, and a significant difference between the two groups was identified [*t*(203) = −3.5899, *p* < 0.01] (Figure 4).

## Discussion

This is the first study characterizing *K. pneumoniae* from poultry sources in Norway, using both phenotypic and genotypic methods. Approximately 40% of the samples were *K. pneumoniae* positive, with significantly higher occurrence in turkeys compared to broilers. A low occurrence of antimicrobial resistance was detected, but interestingly, a hypervirulent clone with a novel ST was detected from turkeys, suggesting a clonal expansion of this lineage in the turkey production in Norway.

The prevalence of *K. pneumoniae* was nearly three times higher in turkeys than in broilers. The samples investigated in this study originated from NORM-VET, and the sampling in the monitoring program is performed to ensure representativeness of the national animal populations. We investigated more than 500 pooled samples and sampling was conducted throughout the whole year. The higher occurrence of *K. pneumoniae* in turkey indicates that turkey is a larger and more important reservoir for *K. pneumoniae* than broiler. This makes turkey an interesting candidate for further study regarding zoonotic transmission. There are many potential factors to why there is a distinct difference in detection rates between the two poultry species. Diet, environment, genetics and the use of antimicrobial agents all influence gut microbiota and could therefore influence the presence of *K. pneumoniae* (Torok et al., 2011). Broiler and turkey reared in large-scale food production have similar living conditions and diet. A possible explanation for the difference in occurrence may be preventive treatment with the ionophores narasin and monensin. In Norway, broilers were previously given narasin and turkey flocks are given monensin as a preventive measure to reduce intestinal parasites (*Eimeria spp.*) (NORM/NORM-VET, 2015, 2017). Currently, turkey flocks are still given monensin, while narasin has been phased out since 2016 in Norwegian broiler production. Ionophores may affect the gut microbiota, as both narasin and monensin have been found to have an antimicrobial effect on gram-positive bacteria (Chan et al., 2020). A reduction of the gram-positive fraction of the gut microbiota may result in higher abundance of Gram-negative bacteria, such as *K. pneumoniae.* Since turkey flocks are still treated with monensin, such preventative treatment may be an explanatory factor for the observed occurrence of *K. pneumoniae* among turkeys in Norway. However, further studies are needed to deduce the effects of such treatment on the gut microbiota. Similarly, antimicrobial treatment may also affect the gut microbiota and change the competition dynamics in the gut for *K. pneumoniae.* In Norway, the use of antibiotics in livestock is low, strictly controlled, and monitored (European Medicines Agency, European Surveillance of Veterinary Antimicrobial Consumption, 2020). In other countries, however, it is used more liberally, not just to combat infections but also as a growth enhancer (Ryan, 2019; European Medicines Agency, European Surveillance of Veterinary Antimicrobial Consumption, 2020). Animals have different microbiomes in their gastrointestinal-tract and excessive use of antibiotics could affect the prevalence of *K. pneumoniae*. The gut microbiome of broiler and turkey is dominated by *Firmicutes* and *Bacteroidetes*, followed by *Proteobacteria, and Actinobacteria* (Oakley et al., 2014; Wilkinson et al., 2017). Although these bacteria belong to the same phylum, a closer look at which species are present and their relative abundance, might give insight into whether they contribute to the variation of *K. pneumoniae* abundance observed in broiler and turkey.

The occurrence of antimicrobial resistance among obtained isolates in this study was low and this reflects the low antimicrobial usage pattern in Norwegian livestock production (European Medicines Agency, European Surveillance of Veterinary Antimicrobial Consumption, 2020). Antimicrobial usage data specifically for broilers also show that the Norwegian production is almost free from selection pressure as only a few broiler flocks are treated annually (NORM/NORM-VET, 2018). The antimicrobials used are mainly phenoxymethylpenicillins and amoxicillin (NORM/NORM-VET, 2018). The finding of low resistance rates is therefore not surprising.

The phylogenetic tree based on poultry genomes comprised several deep-branching lineages, which is typical for *K. pneumoniae* (Wyres et al., 2020). These isolates comprise a set of diverse *K. pneumoniae*, represented by many STs in both animal species. Many of these STs, such as ST13, ST17, ST35, ST37 and ST290, have previously been identified in humans (Marcade et al., 2013; Safavi et al., 2020; Raffelsberger et al., 2021). A recently published study from Norway screened 2,975 healthy persons for *K. pneumoniae* gut carriage (Raffelsberger et al., 2021), and identified a diverse population of *K. pneumoniae*. The study also identified ST35 and ST37 among the most common STs identified in healthy Norwegians, although the occurrence of these STs were found to be only 1.9% each (Raffelsberger et al., 2021). In the current study, ST35 represented a major ST identified in both poultry species. Compared to human carriage, the occurrence of ST35 in Norwegian poultry seem to be higher. Due to the detection of *K. pneumoniae* ST35 and ST37 in both healthy humans and poultry in Norway, it would be of interest to investigate the potential zoonotic aspect further in future studies.

No virulence genes coincided with resistance genes in this dataset, which is typical for *K. pneumoniae* (Wyres et al., 2020). Virulence genes were detected in a relatively high fraction of the genomes, 23.2%. In the Norwegian study on healthy humans as carriers of *K. pneumoniae*, the detection rate of virulence factors was 11.6% (Raffelsberger et al., 2021). There could be several reasons for the lower rate in humans, but in the human study other phylogroups in addition to *K. pneumoniae sensu stricto* were present (Raffelsberger et al., 2021). *K. pneumoniae sensu stricto* has been found to be more strongly associated with virulence than other subspecies (Holt et al., 2015). In contrast, the population in the present study comprised only *K. pneumoniae sensu stricto*, which may have affected detected occurrence of virulence genes. Our findings indicate that poultry may be a reservoir for hypervirulent *K. pneumoniae* populations or the genes encoding virulence. The identified ST4710 clone from turkeys is of particular concern, as all the ST4710 genomes harbored genes that indicate a hypervirulent phenotype, located on a potentially conjugative IncF plasmid (Huynh et al., 2020). These virulence operons, notably aerobactin and salmochelin, are highly important virulence determinants. Plasmids with *iuc5* and *iro5* have previously been identified in *E. coli* (Liu et al., 2015; Lam et al., 2018), which indicate that the plasmid may be transferable. However, this needs to be verified with conjugation experiments. The source of the plasmid and/or the ST4710 clone in the poultry production is currently unknown. The clonal spread of ST4710 indicates a common source. Poultry breeding has a pyramidal structure, which may have contributed to introduction of a specific clone *via* breeding/parent animals with subsequent dissemination downward in the production chain. Introduction *via* breeding animals is believed to be the reason for the occurrence of *E. coli* resistant to 3rd generation cephalosporins or quinolones in the Nordic broiler production (Mo et al., 2016; Myrenås et al., 2018; Kaspersen et al., 2020). The latter results are only based on samples from broilers, therefore further studies are needed to look for the same pattern in the turkey production.

In the current study, almost all tested *K. pneumoniae* were regarded as good biofilm producers. These findings correlate with the previous results from *K. pneumoniae* isolated from humans (Francolini et al., 2017). Additionally, a statistically significant difference in OD_595_ between broilers and turkeys was detected. However, the difference in mean OD_595_ between the two animal species was small and therefore probably not of biological significance. The biofilm producing ability of the isolates were tested at 20 ± 1°C. The good biofilm formation displayed at this temperature indicates that *K. pneumoniae* may produce biofilm reservoirs in both livestock and food production environments contributing to cross contamination of animals as well as products. The core temperature of poultry is approximately 41°C (Purswell et al., 2012). While the isolates in the current study presented good biofilm forming abilities at room temperature, it is unknown if the same level of production efficiency will be observed at body temperature of poultry.

Overall, a higher occurrence of *K. pneumoniae* was detected in turkeys compared to broilers, suggesting turkey as a more important reservoir. Our data indicate a low occurrence of antimicrobial resistance in *K. pneumoniae* in the Norwegian poultry production. However, the presence of a hypervirulent *K. pneumoniae* strain with a potentially transferrable plasmid circulating in the Norwegian turkey production may be cause for concern. In addition, identification of STs found in both animals and in humans may indicate transmission between the reservoirs, but, further studies are required to evaluate the zoonotic potential of these STs. Taken together, the results from this study provides further understanding and knowledge about *K. pneumoniae* in the Norwegian poultry production.

## Data Availability Statement

The raw data used in this study have been uploaded to the European Nucleotide Archive under BioProject PRJEB44689. The p4710 plasmid is available in GenBank under the accession number MW316656. The data used in this study can be found in Supplementary Data Sheet 1.

## Author Contributions

MS conceptualized the study. FF-A, MS, AT, TL, and R-JB did the laboratory work. HK, FF-A, MH, AT, TL, and MS analyzed the data. LN oversaw the biofilm work. All authors contributed to writing and editing the manuscript.

## Conflict of Interest

The authors declare that the research was conducted in the absence of any commercial or financial relationships that could be construed as a potential conflict of interest.

## Publisher’s Note

All claims expressed in this article are solely those of the authors and do not necessarily represent those of their affiliated organizations, or those of the publisher, the editors and the reviewers. Any product that may be evaluated in this article, or claim that may be made by its manufacturer, is not guaranteed or endorsed by the publisher.

## Footnotes

1
https://www.eucast.org/fileadmin/src/media/PDFs/EUCAST_files/Breakpoint_tables/v_11.0_Breakpoint_Tables.pdf

2
https://www.bioinformatics.babraham.ac.uk/projects/fastqc/

3
https://www.bioinformatics.babraham.ac.uk/projects/trim_galore/

4
https://nanoporetech.com/

5
https://github.com/nanoporetech/qcat

6
https://github.com/rrwick/Filtlong
