# Supplementary material for: Exploring Klebsiella pneumoniae in Healthy Poultry Reveals High Genetic Diversity, Good Biofilm-Forming Abilities and Higher Prevalence in Turkeys Than Broilers
Source: Front Microbiol. 2021 Sep 7;12:725414. doi: 10.3389/fmicb.2021.725414 (PMC8453068; doi:10.3389/fmicb.2021.725414)
Supplement: Supplementary file 1 [file Data_Sheet_1.zip › Table 1.DOCX]

Supplementary Material

# Supplementary Data

The file contains the European Nucleotide Archive accession numbers, biofilm- and Kleborate results for all isolates.
